# When a Neonate Is Born, So Is a Microbiota

**DOI:** 10.3390/life11020148

**Published:** 2021-02-16

**Authors:** Alessandra Coscia, Flaminia Bardanzellu, Elisa Caboni, Vassilios Fanos, Diego Giampietro Peroni

**Affiliations:** 1 Neonatology Unit, Department of Public Health and Pediatrics, Università degli Studi di Torino, 10124 Turin, Italy; alessandra.coscia@unito.it; 2 Neonatal Intensive Care Unit, Department of Surgical Sciences, AOU and University of Cagliari, SS 554 km 4,500, 09042 Monserrato, Italy; elisacaboni9@gmail.com (E.C.); vafanos@tin.it (V.F.); 3 Clinical and Experimental Medicine Department, Section of Pediatrics, University of Pisa, Via Roma, 55, 56126 Pisa PI, Italy; diego.peroni@unipi.it

**Keywords:** neonatal microbiota, microbiome, placenta, delivery, breastfeeding, neonatal nutrition, perinatal programming

## Abstract

In recent years, the role of human microbiota as a short- and long-term health promoter and modulator has been affirmed and progressively strengthened. In the course of one’s life, each subject is colonized by a great number of bacteria, which constitute its specific and individual microbiota. Human bacterial colonization starts during fetal life, in opposition to the previous paradigm of the “sterile womb”. Placenta, amniotic fluid, cord blood and fetal tissues each have their own specific microbiota, influenced by maternal health and habits and having a decisive influence on pregnancy outcome and offspring outcome. The maternal microbiota, especially that colonizing the genital system, starts to influence the outcome of pregnancy already before conception, modulating fertility and the success rate of fertilization, even in the case of assisted reproduction techniques. During the perinatal period, neonatal microbiota seems influenced by delivery mode, drug administration and many other conditions. Special attention must be reserved for early neonatal nutrition, because breastfeeding allows the transmission of a specific and unique lactobiome able to modulate and positively affect the neonatal gut microbiota. Our narrative review aims to investigate the currently identified pre- and peri-natal factors influencing neonatal microbiota, before conception, during pregnancy, pre- and post-delivery, since the early microbiota influences the whole life of each subject.

## 1. Introduction

The term “microbiota” defines the whole set of microorganisms that colonize organs and tissue of an individual from the beginning to the end of their life [1] and also persisting after death with the establishment of postmortem microbial communities also called “thanatomicrobiome” [2,3,4].

Placenta, amniotic fluid and fetal tissues, such as skin, lung and gastrointestinal tract, are colonized by these microorganisms since prenatal life [5,6,7,8].

Over the past decade, the human microbiota has been recognized as a new entry in human health; its importance is defined by numerous aspects, allowing us to classify it as a “new organ”. Microbiota’s essential role is determined by its ability to support the biochemical, metabolic and immunological balance of the host organism, necessary for health maintenance [9].

Since birth, our immune system is predisposed to distinguish and destroy invading microbes, and in this context, the human microbiota plays a fundamental role in preventing the growth of pathogens and modulating immunity pathways [1].

Throughout one’s life, microbiota can be influenced and modified by various factors, including maternal health [10,11,12], pregnancy complications, peripartum antibiotic administration [13], mode and place of delivery [14] and breastfeeding [11,15,16,17,18,19,20].

Before conception, female genital tract microbiota seems to influence fertility, pregnancy outcome, post-abortion infection rate and the success rate of assisted reproduction technologies (ART), including embryo-transfer (ET) [21,22,23,24,25,26,27]. The Human Microbiome Project allowed us to expand our knowledge on the characterization, physiology and significance of the microbiota in multiple body sites, as well as on its relationship with the host [28,29].

One of most intriguing themes is the "sterile womb" paradigm, which has been analyzed, during the last ten years, in many studies reporting the presence of bacteria even in sites traditionally considered sterile (uterus, placenta, amniotic fluid, fetus), in physiological conditions as well [5,30]. Even for placenta, the idea of the “sterile” fetus is already outdated [5,6,8].

As is well established in the literature and discussed in this paper, the human microbiota, due to complex and continuous interactions with the host, affects health as a whole and can contribute to the onset of many pathological conditions, even chronic ones. A particularly important function is that performed by the intestinal microbiota, which hosts the most abundant bacterial population.

The purpose of this narrative review is to investigate what the pre- and peri-natal factors are influencing neonatal microbiota, before conception, during pregnancy, pre- and post-delivery.

Much progress has been made, to date, regarding sample collection techniques for the study of the microbiota and for the analysis of bacterial species. Although a detailed discussion of these advances and these novel techniques is beyond the scope of our narrative review, which has a purely clinical purpose, in the literature, very recent papers review sampling techniques for both the gut microbiota [31] and the female genital microbiota [32], as well as the techniques of isolation and culture of the microbiota [33].

## 2. Female Tract Microbiota

### 2.1. Vaginal Microbiota and Fertility

The female urogenital tract microbiota represents only 9% of the whole human microbiota, while that of the gastrointestinal tract represents about 29% [28,34,35].

Thanks to the Human Microbiome Project, we know that the physiological vaginal microbiota is characterized by a relatively low degree of microbial diversity, with the predominance of *Lactobacillus* spp. The vaginal microbiota can be classified into five groups (I–V), a.k.a. “community state types” (CST), based on the presence and types of *Lactobacilli*: CST I (*Lactobacillus crispatus* predominant), CST II (*Lactobacillus gasseri* predominant), CST III (*Lactobacillus iners* predominant) and CST V (*Lactobacillus jenseri* predominant). CST IV is characterized by the presence of non-*Lactobacillus* spp., such as *Prevotella* spp., *Gardnerella* and other bacteria (*Corynebacterium, Atopobium, Megasphera, Sneathia*) [28,36,37]. Successively, CST IV was further divided into type IV-A characterized by low proportions of *Lactobacillus iners* or other *Lactobacillus* spp.; various species of anaerobic bacteria including *Anaerococcus*, *Corynebacterium*, *Finegoldia* or *Streptococcus*; and type IV-B, showing a higher proportion of the genera *Atopobium*, *Prevotella*, *Parvimonas*, *Sneathia, Gardnerella, Mobiluncus, Peptoniphilus* and several other taxa [38].

During the healthy reproductive life and during pregnancy, the composition of vaginal microbiota changes according to the cyclic fluctuations of estrogen and progesterone levels. However, the variations in composition are slight and only consist of a relative predominance of one lactic acid-producing bacterium over another. In fact, estrogen and progesterone both help to ensure adequate availability of glycogen, metabolized by *Lactobacillus* spp., into lactic acid, which guarantees the normal acid vaginal pH [37,38,39,40,41].

The presence of *Lactobacilli* and a normal vaginal acid pH protect against a possible pathological growth of anaerobic species, such as *Gardnerella vaginalis*, *Mycoplasma hominis*, *Atopobium vaginale* and *Mobiluncus curtisii*. These bacteria prevail in the so-called bacterial vaginosis (BV), which is characteristic of pre-menopausal age and pathological conditions [42,43,44,45]. BV is well known to be associated with adverse outcomes in obstetrics and gynecology, such as preterm birth and post-surgery infections [21,22,23,24]. On the other hand, there are only a few studies concerning the relationship between the female genital tract microbiota and infertility.

The Human Microbiome Project demonstrated that the vaginal microbial diversity is very low in comparison to other sites (e.g., oral cavity), with a higher diversity being associated with BV [29,46].

Usually, in a microbial ecosystem, a high biodiversity is synonymous with health, while a significant decrease in biodiversity is defined as a status of dysbiosis, associated with several pathologies [47,48,49]. The unique exception is the vaginal ecosystem, dominated by *Lactobacilli*, where high biodiversity is linked to an unhealthy status, as reported above [29,46].

Two metanalyses pointed out that 19% of infertile patients had BV; on the other hand, according to the same metanalyses, BV does not significantly impair conception rate but increases the rate of early pregnancy loss [50,51]. The analyzed studies also show the association between anomalies in the vaginal microbiota and tubal infertility, probably due to the ascent of pathogens through the cervix (e.g., *Chlamydia trachomatis*), triggering inflammation [50,51,52,53].

However, all these studies were performed using the classical culture-based technology and the so-called Nugent score, based on the bacterial classification by Gram staining [26,54]. Culture-based technology has significant methodological limitations: some bacteria cannot be cultured nor identified; moreover, it can be difficult to distinguish the bacteria from each other. These limitations lead to a risk of both underestimating and overestimating the presence of pathogenic bacterial species [55,56]. Recently, sequencing and metagenomic methods have considerably enriched our knowledge on the relationship between vaginal microbiota, infertility and the outcome of pregnancies from ART.

The study carried out by Campisciano and colleauges showed that, comparing infertile women to fertile ones, *Lactobacillus gasseri, Veillonella* spp. and *Staphylococci* were over-represented, while *Lactobacillus iners* and *Lactobacillus crispatus* were under-represented [57].

The composition of the vaginal microbiota also impacts the outcome of ET. Hyman et al. demonstrated that the probability of a live birth is related to the diversity of species and to the presence of *Lactobacilli* on the ET day [58]. Other authors [25,26] reported the negative (although not statistically significant) effect of BV on the implantation rate.

### 2.2. Uterine Microbiota and Fertility

Traditionally, it was believed that the uterine cavity was sterile, and bacterial colonization was considered a pathological finding [59]. However, the existence of an intrauterine microbiota, characterized by remarkable stability between the follicular and luteal phase, was only recently demonstrated [60].

Mitchell et al. [52] confirmed that the upper genital tract is not sterile, uncovering the presence of at least one bacterial species in that site.

The bacteria located in the endometrial cavity and in the upper part of the cervix resemble those present in the vagina (*L. iners, L. crispatus, Prevotella* spp.), albeit in a smaller quantity (about 4 times less), although many more bacterial species are present in the vagina [61]. The relative bacterial scarcity in the uterine cavity, compared to the vaginal environment, could be due to the partial barrier action carried out by the endocervix or to the endometrial immune response [52].

One of the biggest criticisms aimed at these findings is the possible contamination during the collection of the uterine samples by the cervico-vaginal microbiota. However, Chen et al. showed a high degree of similarity between the uterine microbiota collected directly by surgery and that collected trans-cervically [61]. On the contrary, in a very recent study, the samples were taken with a particular method based on the combined use of two specific catheters and accurate tissue disinfection; thus, the procedure could be considered almost sterile. The absent contamination by the vaginal flora, as a result, highlighted a characteristic heterogeneous endometrial microbiota (also including newly identified genital bacteria such as *Kocuria dechangensis* and the absence of *Lactobacilli)* different from the vaginal one (dominated by the *Lactobacillus genus)* [62]. Although interesting, these results should be confirmed in future studies based on the same sampling technique.

The uterine microbiota is also likely to affect fertility [63]. Using traditional bacterial cultures, many authors demonstrated an association between the presence of pathogenic endometrial bacteria from the ET catheter and low pregnancy rates after ART [64,65,66,67,68]. The presence of pathogenic bacteria was shown to decrease with the preventive use of antibiotics [65].

In the last decade, the use of next-generation sequencing of bacterial 16S rRNA gene provided a better characterization of the microbiota during ART and allowed 278 genera to be isolated, among which *Lactobacillus* spp. and *Flavobacterium* spp. are predominant [69]. Another larger study conducted by Moreno et al. identified two microbiota profiles, one of which is *Lactobacillus* spp.–dominated (LD), while the other is non-*Lactobacillus* spp.–dominated (NLD): the latter has been associated with a lower implantation rate [60].

On the contrary, according to Riganelli et al., endometrial colonization by vaginal flora, especially *Lactobacillus species* by translocation, seems to have a negative impact on the outcome of ART [62], suggesting that the subject, still characterized by controversies, deserves clarification through future studies.

Other authors used mRNA analysis to identify less abundant bacteria, and therefore isolating, in addition to *Lactobacillus* spp., also *Corynebacterium* spp., *Bifidobacterium* spp., *Staphylococcus* spp. and *Streptococcus* spp. However, the authors did not make any comparison with traditional culture techniques [70].

It has not been clarified through which mechanisms the microbiota influences the implantation rate. It has been speculated that a positive action of *Lactobacillus* spp. could be mediated by the acidification of vaginal pH, which inhibits pathogenic bacteria: however, no difference was found between endometrial microbiota and endometrial pH. Instead, an abnormal endometrial microbiota could trigger an inflammatory cascade with detrimental effects on the implantation. This hypothesis needs to be supported by further studies [50,71].

At present, the results of the studies (including meta-analyses) concerning the relationship between microbiota and fertility in ART, while suggesting a negative influence by an abnormal microbiota, do not allow definitive conclusions. Further studies would be needed, with adequate sample size and comparison between new sequencing methods and traditional culture techniques. Interventional studies are also lacking, especially considering the ethical problems related to them; however, coming from the assumption that the composition of the microbiota influences fertility, it would be highly useful to identify how to modify it and therefore to demonstrate whether these interventions could be effective [50,51,71].

In Table 1, major bacterial taxa found at each colonization site of reproductive age women, and their impact on fertility, are reported.

## 3. Microbiota and Pregnancy

Pregnancy produces a series of changes involving the entire maternal and fetal dyad [72,73,74]. The maternal microbiota also experiences changes in the various sites (gut, oral cavity, vagina); the findings are not homogeneous because of the wide variability of characteristics of populations included in the studies (ethnicity, gestational age-GA, geographic and environmental factors, lifestyle habits) [75,76,77]. 

There are many factors influencing maternal microbiota changes, such as maternal diet [78,79,80,81], pre-pregnancy weight, weight gain and some pathological conditions, such as diabetes and obesity [82,83,84,85]. During pregnancy, and especially in the third trimester, the maternal gut microbiota experiences a reduction in bacterial diversity, with an increase of *Proteobacteria*, *Streptococci* and some specific *Lactobacilli* types: this composition, necessary and beneficial for the normal course of pregnancy, highlights host–microbial interactions that impact host metabolism. Specifically, insulin resistance is increased, promoting energy storage for fetal growth. However, the future implications of these metabolic changes on maternal and fetal health are mostly unknown [75].

In Table 2, we summarized the major bacterial taxa found at each colonization site during pregnancy and its complications.

### 3.1. Physiological Changes in Pregnancy

The effects of hormonal changes during pregnancy (increased estrogen and progesterone levels) are different according to the site of action, altering in a different way, for example, the gut microbiota rather than the oral microbiota. The gut microbiota, during pregnancy, is characterized by a low alpha diversity index (representing within-sample phylogenetic diversity) [45,75,95] and a high beta diversity index (representing a measure of the evolutionary distance between microbiota), while the oral microbiota, during the third trimester, experiences an increase in the amount of bacteria and of alpha diversity [75,89,90]. At the intestinal level, microbiota composition varies throughout the progress of pregnancy: during the first trimester, it is very similar to that of non-pregnant fertile women; subsequently, *Bifidobacteria*, *Proteobacteria* and bacteria producing lactic acid prevail [86].

The vaginal microbiota also undergoes many changes: as the GA increases, there is a reduction in anaerobic bacteria and an increase in particularly stable *Lactobacillus* spp., which are able to guarantee adequate protection against pathogens dangerous for the outcome of pregnancy [91,92].

Traditionally, the pregnant uterus was considered a sterile environment in defense of the fetus, and any bacterial colonization was considered a pathological condition [45]. The infant microbiota has therefore always been thought of as acquired by the newborn during birth and subsequently horizontally through contact with the mother and the environment.

This theory has been contested in the last decade, after some authors, in 2011 [96], highlighted the presence of bacteria in sites once considered sterile (placenta, amniotic fluid, meconium). The reversal of the theory drew much interest, to the point of deserving an article published in ‘Nature’ in 2018 [6], which analyzes the history of controversies on this issue.

The Human Microbiome Project significantly boosted the research on placental microbiota: one of the participants in the project (Aagaard and his team), in 2014, reported a discrepancy between the microbiota of newborn babies during the first week of life and that of the vagina of pregnant women, thus proposing the acquisition of the microbiota during birth and hypothesizing a bacterial transfer through the placenta [5,97].

In order to study placental colonization while avoiding contamination, Aagaard conducted a study on 320 women (one group had physiological pregnancies, while the other presented pathological conditions such as prematurity or infections) by collecting placental samples with sterile methods, using comparative next-generation sequencing of bacterial 16S rRNA gene and whole-genome shotgun (WGS) metagenomic technique and controls to rule out contamination. Many placental samples contained bacterial DNA: by sequencing the whole genome, a prevalence of *Escherichia coli* has been shown [5]. 

Aagard and colleagues [5] also compared the placental microbiota with that from other sites, discovering that the main similarities were found with the oral microbiota: one of the hypotheses is that bacteria can reach the placenta by a hematic route. Many other authors reported the presence of small quantities of bacteria in the placenta of healthy women [98,99,100,101,102], in particular *Lactobacilli*, *Propionibacteria* and *Enterobacteriaceae* [30], by using both culture methods and metagenomics. 

Further elements in favor of the prenatal colonization theory are the numerous studies reporting the presence of bacteria in the amniotic fluid and in the umbilical cord, also in physiological situations [93,98,103,104,105,106,107,108,109,110,111,112,113,114,115]. Other data supporting in utero exposure derive from studies conducted on meconium, which have detected the presence of a microbiota also in this site, traditionally considered sterile [7,100,101,116,117]. 

The meconial microbiota is characterized by a low alpha diversity index and a high beta diversity index [118,119]: the most represented species were *Enterobacteriaceae, Enterococcus* spp., *Lactobacillus* spp. and *Bifidobacterium* spp. [117,120]. Recently, Tapiainen at al. [121] affirmed, using a series of 218 infants, that the presence of maternal factors (such as consumption of probiotics or the presence of furry pets at home) during pregnancy, and even mode of birth/drug administration during delivery, can influence the composition of the meconium microbiota. 

Literature data have shown that the composition of meconium microbiota is very similar to that of the amniotic fluid [122], even within mother–fetus pairs [93]: the fetal intestine could therefore become colonized thanks to the continuous ingestion in the uterus of small quantities of amniotic fluid. This route of transmission was demonstrated to be possible by Jimenez et al., using a murine model: after the oral administration of *Enterococcus* spp. to the mother, the same bacterium was found in the gut of the pups delivered by Cesarean section (CS) [116].

However, it is still unclear when and how the in utero exposure takes place: it is possible for the fetus to get colonized through multiple routes, which include the ascent from the vagina and the hematic route through the placenta from the oral cavity, the urinary tract or the gut. It is not completely clear whether the fetal intestine is only a "passive spectator" who witnesses the mere passage of microorganisms or whether it constitutes an active milieu in which the bacteria can grow, reproduce and take on a biological role.

Dominguez-Bello [123] stated that the results relating to the meconium microbiota do not have a single interpretation. In fact, the emission of the first meconium generally takes place a few hours after birth: in this time window, there are many opportunities for the newborn to get in touch with the maternal microbiota outside the uterus (labor, passage in the birth canal and contact with maternal skin, including in the case of CS).

Perez-Munoz, in 2017, conducted a critical assessment of both the "in utero colonization" and the "sterile womb" hypothesis and concluded that the few pieces of evidence that actually had a high methodological quality supported the second hypothesis more [115]. In fact, the only well-controlled study [124] that analyzed the oral, vaginal and placental microbiota, also using contamination controls, concluded that it was not possible to identify a characteristic placental microbiota, as there were no differences between placental and control findings. 

The main difficulties found while interpreting these data are caused by the methodological limits regarding the techniques used. While at oral, intestinal and vaginal levels the quantities of bacteria are abundant, in the placenta and in the amniotic fluid, small quantities can be found. The main risks are those deriving from the use of techniques with low detection sensitivity and from the lack of adequate controls. In fact, the possibility of contamination in the reagents (DNA extraction kit and PCR reagents) is a big problem where the bacterial load is very low (placenta, amniotic fluid, meconium) [125,126].

Thus, more accurate contamination risk reduction techniques and full-length 16S rRNA gene sequencing methods could be the most appropriate to analyze the amniotic fluid and the meconium microbiota [125,126,127,128,129]. This way, Stinson et al. [128,130] showed bacterial DNA in all meconium samples and in most of those of amniotic fluid in 50 women undergoing elective CT and in their newborns. Using the same samples, they also analyzed the levels of inflammatory cytokines and immunomodulating short-chain fatty acids (SCFAs): the levels of acetate and propionate present in all meconium samples were similar to those reported in previous studies in children, suggesting that gut microbiota would seem to play an active metabolic role since the early phases of life. Cytokine levels in the amniotic fluid correlated with the composition of the amniotic fluid microbiota [128,130].All these findings contribute to supporting the hypothesis that colonization in the uterus is possible. These data acquire an even more important value if we consider that microbiota at birth will influence the immune system of the newborn during its development [1,131,132,133].

Furthermore, DNA sequencing techniques do not allow us to differentiate between live active bacteria and dead inactive ones. It will be necessary, for future studies, to use a combination of the available techniques (culture-based, sequencing-based) and to utilize new methodologies (metagenomics, metabolomics, proteomics, metatranscriptomics) to obtain a more detailed characterization of the bacterial species that colonize a given site, of any sequences of bacterial derivation and of metabolites deriving from the bacteria or from the host itself that will be able to dynamically describe the interactions between the microbiota and its host [128,130]. 

### 3.2. Microbiota in Pathological Pregnancies

Maternal gut dysbiosis, during the third trimester of pregnancy, together with changes in the function of the mucosal immune system, could cause an increase in the epithelial permeability to glucose, potentially conditioning maternal metabolism and therefore the fetal transfer of nutrients [75,134,135].

One of the most studied associations is between the composition of maternal microbiota and prematurity or low birth weight. The relationship between the growth of pathogenic bacteria or BV and the risk of miscarriage or preterm birth is well known [77]. Other studies have shown that, during the third trimester, less richness and less diversity in the vaginal microbiota are associated with a higher risk of preterm birth, to the point of proposing certain microbiota anomalies as diagnostic markers [136,137,138,139].

Not only maternal microbiota was shown to be altered in premature deliveries. In fact, the preterm infant also presents a different meconial microbiota compared to that of the term infant [122,140,141], and there is a correlation between low GA and lower bacterial diversity [122,140]. In particular, in the meconium of preterm infants born by mothers with chorioamniotitis, there are large quantities of pathogenic bacteria, such as *Ureaplasma parvum, Fusobacterium nucleatum* and *Streptococcus agalactiae* [30].

The most represented species in newborns < 33 weeks of GA are *Lactobacillus* spp., *Staphylococcus* spp., *Enterobacter* spp. and *Enterobacteriaceae* [122,141]. The intriguing hypothesis is that some bacteria may induce the fetal intestine to produce and release pro-inflammatory proteins that are involved in preterm labor; it is interesting to note that some of these proteins have been also found in amniotic fluid [106,122].

Every maternal factor (physiological, pathological or environmental) can influence the composition of the microbiota, and therefore health in a broader sense. If the hypothesis of a uterine microbial colonization is true, numerous maternal factors acting during the pregnancy and the peri-postpartum period can affect the composition of the fetus-neonatal microbiota and therefore the future infant health [100]. Some studies described the influence of maternal diet during pregnancy on the composition of meconial microbiota. Chu et al. [142] reported a lower percentage of *Bacteroides* in babies of 81 women following a high-fat diet, while Lundgren et al. [143] analyzed 145 infant/mother pairs, highlighting that the composition of the maternal diet influences the infant’s fecal microbiota and that one of the main variables is the delivery mode (spontaneous or elective CS).

Maternal microbiota also changes in relation to pre-gravidic weight and weight gain in pregnancy: in women who are overweight or undergoing excessive weight gain, there is a reduction in *Bifidobacterium* spp. and *Bacteroides*, and an increase in *Enterobacteriaceae, Staphylococcus* spp. and *Escherichia coli* [84,87]. These alterations can condition the fetal and neonatal microbiota (also through breastfeeding), with an increase in *Bacteroides* and a reduction in *Enterococcus* spp., *Acinetobacter* spp. and *Pseudomonas* spp. [20,144,145], and the influence of maternal weight on neonatal microbiota acquires more relevance if we consider the large incidence of overweight/obesity among pregnant women, which is around 30% in Europe [146].

Moreover, maternal conditions such as dysbiosis, preeclampsia and gestational diabetes mellitus (GDM) have been identified as causes of premature birth or fetal adverse outcome, including necrotizing enterocolitis (NEC), late-onset sepsis and, successively, food intolerance, with mechanisms potentially involving maternal/fetal microbiota [12]. A Chinese study investigating 100 pregnant women in different stages of pregnancy evidenced a higher percentage of pathogenic bacteria, such as *Clostridium perfringens* and *Bulleidia moorei*, and a reduction in the *Coprococcus catus* in the gut microbiota of mothers affected by preeclampsia, while healthy controls were mostly characterized by *Bacteroidetes* spp. [88]. From this study, the authors concluded that these microbiological characteristics associated with preeclampsia may become new markers for such conditions [88].

GDM was also studied as a potential factor influencing maternal/neonatal microbiota; placental microbiota of women with GDM was recently investigated in relation to maternal metabolism and placental expression of anti-inflammatory cytokines, such as IL10, TIMP3, ITGAX and MRC1MR. The results of Bassols et al. showed a higher percentage of *Acinetobacter* spp. in women with GDM; moreover, the abundance of such a type of bacterium can also influence metabolic and inflammatory phenotype [94]. These results suggest that the placental microbiota may be a possible new therapeutic target in GDM [94].

Moreover, metabolic hormone levels and microbiota profiles were found different by comparing overweight and obese women and hormone levels correlated with specific microbial changes [85]. In this study, a relationship occurred between fecal microbiota profile and maternal circulating insulin, C-peptide, glucagon, incretin and adipokine by comparing overweight (n = 29) and obese (n = 41) pregnant women at 16 weeks of gestation. As a result, adipokine levels strongly correlated with *Ruminococcaceae* spp. and *Lachnospiraceae* spp., involved in energy metabolism, and insulin was positively related to the *Collinsella* spp. Gastrointestinal polypeptide was positively correlated with *Coprococcus* spp. but negatively correlated with the *Ruminococcus* spp. This study showed new relationships between gut microbiota, maternal weight and hormone levels, suggesting that manipulation of gut microbial composition could influence maternal metabolism during pregnancy [85]. 

Finally, in a study carried out on 64 women aiming to evaluate whether women with GDM can be treated with probiotics, it was found that one probiotic capsule/day for 8 weeks can improve glucose metabolism and reduce weight gain. Probiotics seem to balance material microbiota, normalize gut permeability, regulate inflammatory mediators and control energy metabolism [147].

## 4. Impact of Maternal–Fetal Microbiota on Development

It is likely that maternal and fetal microbiota, interacting with each other, can exert a fundamental effect on fetal growth in general and in particular on the development of the immune system and nervous system [74,100,114].

The influence of the maternal microbiota is probably exerted by two mechanisms. First, the maternal intestinal microbiota can act directly on growth and development processes, in particular of the immune and nervous systems, through the production of metabolites that can reach the fetus through the placenta [74,114,148,149]. The second mechanism could be played by the fetal microbiota, especially the intestinal one, which would exert its action on development and programming directly on-site [100,150,151,152].

The action of colonization in utero would therefore be fundamental in determining long-term health, even in adulthood [109,110,112,113]. One of the fundamental actions of bacterial exposure in utero would be to modulate the programming of the immune and metabolic system: the primitive immune system requires interaction with bacteria in order to learn to distinguish the harmful ones from the useful ones [100,153,154,155].

What are the effectors of the immunomodulating action has been the subject of numerous studies in the last decade: an important role is played by SCFAs produced by the microbiota, which can act locally by regulating the production of T-cells and IL-10 [131,132,156] or by inducing an anti-inflammatory action by reaction with metabolite sensing G-proteins coupled receptors (GPRs). However, the SFCAs themselves could also enter the circulation and exert their action at a distance, for example on dendritic cells and on bone marrow macrophages [133]. Other molecules could be implicated in the primer action by the fetal microbiota, such as toll-like receptors (TLR), present on macrophages, dendritic cells, mast cells), capable of recognizing bacterial antigens and therefore influencing the fetal immune system [157,158].

The gut microbiota is also thought to be essential in bi-directional communication between the gastrointestinal tract and the central nervous system. A particularly fascinating hypothesis, studied especially in animal models, concerns the role of maternal and fetal microbiota in the development and functions of the central nervous system (in particular on behavioral aspects). The third trimester of pregnancy, just when the maternal intestinal microbiota becomes more abundant, is also characterized by a greater passage of nutrients to the fetus and constitutes a sensitive phase for the processes of synaptogenesis, myelinization and development of some specific areas, such as the hypothalamus [159,160,161]. The maternal–fetal microbiota, at the center of metabolic processes, is likely to contribute to brain development through mechanisms that are still poorly understood: microbiota-derived metabolites can constitute a substrate for neuronal development, stimulate energy production and induce remodeling and receptor activation [161].

Early childhood disturbances of the developing gut microbiota can impact neurodevelopment and lead to negative mental health outcomes throughout life [162,163,164,165,166,167,168,169]. In addition, some psychiatric diseases of children and adults have been associated with the exposure, during fetal life, to unfavorable factors such as hypoxia and reoxygenation. In response to altered oxygen concentrations, the placenta seems to releases certain substances that damage developing neurons [170,171,172,173]. Thus, brain damage can occur not only due to a lower oxygen supply than required, but also due to the accumulation in the fetal circulation of reactive products, released from the placenta, that negatively affect the vascularization and metabolism of the brain [174].

### 4.1. Neonatal Microbiota

The human gut microbiota is one of the most important environmental factors affecting human health; it plays a relevant role in metabolism, immunity and development and is highly conserved during evolution [175,176,177].

Neonatal gut colonization may be defined as the “de novo” assembly of a bacterial community, and it is influenced by maternal, dietary, clinical and pharmacological factors [178].

During all the phases of pre- and post-natal growth, gut microbiota undergoes modifications in its quality and quantity. Moreover, in perinatal life, the fetus is highly influenced by environmental factors and maternal health conditions [178,179].

In Table 3, we summarized the major bacterial taxa found in newborns at each colonization site.

Alterations involving the intestinal microbiota during the first months of life of babies born by overweight mothers can affect short- and long-term health. In particular, a low amount of gut *Bifidobacterium* spp. can determine a greater degree of inflammation and a greater ability to produce energy from food, leading to an excessive early weight gain during the first months of life. This could represent an important risk factor for the development of obesity in childhood and therefore in adulthood [83,182]. 

Neonatal oral and gut microbiota appears to be influenced by several environmental factors, including mode of birth and breastfeeding, and, generally, *Streptococcus* spp. colonizes neonatal oral cavity early after birth. It has been observed that maternal hygiene habits can also influence the composition of the oral microbiota of the newborn, since the bacteria found in the maternal mouth are very similar to those found in the placenta [5,11,180]. Thus, maternal oral microbiota is fundamental for the formation of neonatal microbiota. It has been also shown that oral infections in mothers can be associated with abortion, altered fetal development or premature birth, through inflammatory responses and immune responses [5,11,180]. 

The host genome controls the first bacteria that will colonize the host even through the variety and availability of adhesion sites. The first colonizers, for their part, influenced by several factors, including delivery mode of breastfeeding, act as pioneers and have the ability to modulate subsequent colonization through the modulation of the expression of receptor sites, which is important for the composition of the final microbiota and therefore for the development of many pathologies in adult age [123,195,196,197]. 

In animal models, *Campylobacter rectus* and *Porphyromonas gingivalis* infection during pregnancy resulted in a significant reduction in maternal fertility, and *Fusobacterium nucleatum* was able to cause fetal death.

Therefore, if the pregnant woman’s oral microbiota is not optimal, dangerous bacteria could be transmitted to the placenta and potentially impair fetal health. In particular, mothers with chronic periodontitis have a high chance of undergoing premature delivery compared to healthy mothers, since bacterial translocation to the placenta determines prostanoids activation, inducing uterine contractions and preterm labor [5,10,11,180]. 

Some preliminary data suggest that one week after birth, neonatal oral biofilm resembles that of the mother. Moreover, the presence of anaerobic Gram-negative *Fusobacterium nucleatum*, associated with chronic periodontitis, was also correlated to BV and preterm births [10,181]. There are some species of bacteria, coming from the oral cavity (e.g., *Fusobacterium nucleatum*), that are capable of being transmitted in a hematogenous way and of modifying the permeability of the vascular endothelium, thus allowing the passage of other microorganisms such as *Escherichia coli* [198]. Furthermore, some authors have highlighted, in healthy women who give birth by elective Cesarean section (CS), the presence of a specific placental microbiota, less abundant, with a lower diversity index and with a prevalence of Proteobacteria [93]. 

Regarding perinatal factors, during delivery, the newborn undergoes a considerable microbial modification, and early microbial colonization triggers processes that influence intestinal and immune maturation [14]. 

Perinatal factors, and especially mode of delivery and the place where it occurs (hospital vs. home), are fundamental for modeling the infant gut microbiota, potentially influencing neonatal outcome.

Mode of delivery is a key factor determining early microbial colonization. Newborns born by vaginal delivery (VAG) acquire microbial communities similar to the maternal gut and vagina; on the contrary, infants born by CS acquire environment-like bacteria, such as *Staphylococcus* spp., *Corynebacterium* spp. and *Propionibacterium* spp.

Infants born by CS are associated with lower microbial diversity, delayed colonization of *Bacteroides* spp. and *Bifidobacteri* and impaired immune responses [14]. 

This is in agreement with a very recent study affirming that newborns delivered by CS lose contact with maternal vaginal microbiota, instead of those born by VAG. Consequently, CS impairs the early establishment and development of the infant gut microbiota. The immature gut microbiota observed in CS infants is associated with adverse outcomes later in life, such as immune and metabolic disorders. In this study, a survey of 132 Korean newborns was carried out; 64 were born by VAG and 68 by CS. All the enrolled newborns received the same postpartum care services up to two weeks. Fecal samples were collected on days 3, 7 and 14; as a result, in the group of infants born by VAG, the abundance of *Bifidobacterium* spp. (7th and 14th day), *Bacteroides* spp. (7th and 14th day) and *Lachnospiraceae* spp. (7th day) was significantly greater than CS infants, with a lower abundance of *Enterobacteriaceae* spp. [194]. This analysis showed that mode of delivery is the major determinant of neonatal gut microbiota; infants born by CS acquire a microbiota more similar to the skin (such as *Propionibacterium* spp., *Staphylococcus* spp. and *Corynebacterium* spp.). Furthermore, it was observed that the fecal microbiota of 72% of neonates born by VAG resembles that of their mothers; in neonates delivered by CS, this percentage is reduced to 41% [194]. 

The neonate born by CS does not pass through the birth canal and becomes mainly colonized by environmental bacteria or colonizing maternal skin, a circumstance that does not happen in VAG [199]. 

During the first year of life, the intestinal microbiota develops according to the diet, and its diversity increases. At about 2.5 years of age, the composition, diversity and functions of the infant microbiota resemble those of the microbiota of adult people [200]. During adult life, intestinal microflora appears to be relatively stable up to 65 years of age, as the microbial community shifts, with the increase of *Bacteroidetes* spp. and *Clostridium* spp. [201]. 

Collado and co-workers tried to justify the impaired immune responses shown by neonates born by CS also with the administration of pre and intra-partum antibiotic and other medical practices performed during CS, potentially interfering with the early gut colonization and predisposing the newborn to develop long-term immune disorders, including asthma, allergy, obesity and diabetes [14]. Thus, it can be deduced that medical interventions before and during delivery are critical for neonatal microbial colonization and for proper maturation of the immune system, which can influence long-term outcome [13,14]. 

CS seems associated with the earlier onset of several diseases in childhood or adulthood, such as pediatric obesity, type 2 diabetes and allergies. Over the past years, some studies have shown that babies born full-term from VAG show a significantly different physiology at birth than those born by CS [202,203]. Martin et al. performed a metabolomics study (proton nuclear magnetic resonance spectroscopy, ^1^H-NMR) on urine samples collected immediately after birth from 42 neonates, with comparable GA and birth weight; urinary samples significantly separated according to delivery mode, since samples from neonates born by CS showed a lower urinary excretion of dicarboxylic acids, compared with samples from VAG babies, highlighting lower oxidation of omega acids [204]. This specific metabolic pathway could be a potential explanation of the current evidence of the lower body temperature at birth shown by babies born by CS, potentially following altered thermogenesis mechanisms. In addition, CS delivery is also associated with hypoglycemic conditions and an altered endocrine profile that involves changes in the metabolic energy pathways. Respiratory function may also be impaired by CS, due to a reduced/delayed surfactant production [204], and metabolomics seems a useful technique to describe such pathways and perinatal conditions affecting neonatal metabolism.

The administration of intra-partum antibiotics seems to have profound effects on the gut colonization of the newborn, both reaching fetal circulation via umbilical cord and even altering maternal vaginal and intestinal microbiota (influencing vertical microbial transmission), and this could lead to a decrease in bacterial diversity in the newborn, with a decrease in the proportion of *Actinobacteria* and *Bacteriodetes* and a simultaneous increase in *Proteobacteria* [13]. 

Gut microbiota is essential in the maturation of the neonatal immune system; factors affecting its equilibrium could lead to short- and long-term onset of pathologies in the offspring such as the increase in early-onset Gram-negative sepsis, bronchopulmonary dysplasia, obesity, asthma, eczema, inflammatory bowel diseases and greater resistance to antibiotics [13].

The most frequently prescribed and administered antibiotics are beta-lactams (ampicillin and penicillin), mostly for the prevention of neonatal group B Streptococcal infection and also useful for preventing maternal morbidity after CS. 

Modifications of neonatal microbiota can also be associated with pathologies in the newborn, including NEC and sudden infant death syndrome (SIDS). In NEC, an abnormal gut colonization by *Pseudomonas* spp. and *E. coli* was detected; moreover, a metabolomics study on urine samples detected an increase in gluconic acid, a bacterial-derived metabolite in the urine of patients with NEC [183,184,185,186,187,188,189,190,191,192]. 

The pathogenesis of SIDS remains an open question, even though a recent hypothesis tried to demonstrate the role of the gut microbiota. A study investigating gut microbiota in 52 infants whose death was caused by SIDS and 102 healthy controls showed higher levels of *Clostridium difficile*, *Clostridium innocuum* and *Bacteroides thetaiotaomicron* in SIDS cases, and autopsy data of infants with SIDS resemble those of septic shock, demonstrating that the intestinal microbiota of SIDS coincides with a proinflammatory state [193]. 

Interestingly, the presence of a specific airway microbiota at birth was also pointed out [205], potentially deriving from fetal life. In fact, the exact moment for such airway colonization is still partially clarified, and it was observed that airway microbiota doesnot significantly differ among neonates born by VAG or CS [205], suggesting a possible placental/uterine colonization.

Al Alam and co-workers firstly investigated human fetal and placental microbiota from 11 to 20 weeks of GA. In this study, microbial DNA was detected in fetal lungs and placenta since 11 weeks of gestation, with a partial overlap among microbial species detected in these two tissues. Moreover, lung microbiota was shown to modify itself during gestation, and such maturation could be determined by maternal or intrauterine factors [206]. These findings could suggest maternal transplacentar transfer of microbial DNA that, reaching the fetus, could trigger his colonization and promote the development of the immune system [206]. 

### 4.2. Human Breast Milk and Microbiota

From an evolutionary and nutritional standpoint, human breast milk (HBM) is the ideal food for the human infant for the first months of life: it is a species-specific food, with a composition designed by nature to better respond to the biological and psychological needs of the newborn. HBM is considered the gold standard nourishment for the infant because of its wide variety of bioactive compounds that change their composition overtime to satisfy the needs of the growing infant [207]. HBM is a blend of immune active factors, oligosaccharides and microbes, which all may influence early immunological outcomes [208]. 

Moreover, HBM contains typical components of the innate immunity that are lacking in an infant’s immature defenses, which protect the infant, dictating additional selection on the infant gut microbiota. In particular, lactoferrin is a protein that binds the iron present in milk, limiting its availability to pathogens and also preventing these bacteria from binding to the intestinal barrier. Lactoferrin interferes with viral anchoring and prevents the subsequent mechanisms that allow the viral concentration on the cell surface, as well as the contact with the specific entry receptors, namely ACE2, that allows the full infection [209,210].

The bacterial load of HBM has a role in the infant gut; it was observed that it contributed to infant digestion, had a protective role competing with pathogens and increased mucine production, reducing intestinal permeability and improving its functions [211]. Therefore, HBM bacterial communities seem to act as a natural prebiotic for infant microbiota, educating the infant’s immune system and offering protection against allergy development later in life [212,213]. HBM microbiota induced an adequate intestinal immune homeostasis that initially promoted a shift from an intrauterine Th2-predominant to a Th1/Th2 balanced response and a stimulation of T-regulatory cells [214]. 

Historically HBM was thought to be sterile and free of microorganisms; the presence of bacteria was attributed to milk contamination after expression or mammary gland infection [215,216,217]. To date, shotgun metagenomics analysis of human milk by total DNA reported that human milk contains >360 prokaryotic genera, with *Proteobacteria* (65%) and *Firmicutes* (34%) as the predominant phyla, and with *Pseudomonas* spp. (61.1%), *Staphylococcus* spp. (33.4%), and *Streptococcus* spp. (0.5%) as the predominant genera [218]. In addition, several yeasts and fungi have been identified in HBM of healthy mothers, including *Malassezia, Candida, Saccharomyces* and *Rhodotorula* [219]. 

A value of approximately 10^6^ cells/mL has been estimated to be HBM bacterial load, indicating that “a breastfed infant feeding 800mL of milk per day would ingest 10^7^–10^8^ bacterial cells daily” [220]. 

Martin et al., at the beginning of 20th century, evidenced commensal and probiotic bacteria in HBM by the use of culture-dependent techniques and found, in all samples, the presence of the lactic acid bacteria *Lactobacillus gasseri* and *Lactobacillus fermentum*. This type of bacteria reduces the growth of potential pathogenic organisms in the gastrointestinal tract thanks to the production of acetate and lactate from the metabolism of the sugars ingested by the host [221]. The development of culture-independent DNA-based techniques including denaturing gradient gel electrophoresis (DGGE), temperature gradient gel electrophoresis (TGGE) and next-generation sequencing (NGS) allowed the detection of new additional bacterial genera in HBM like the obligate anaerobes, particularly *Bifidobacterium* spp., *Bacteroides* spp. and members of the *Clostridia* class *(Blautia, Clostridium, Collinsella* and *Veillonella* spp.) [222]. NGS resulted in the identification of a broad range of microbes common to different body sites, from *Veillonella* and *Prevotella* spp., common to the oral cavity, to the skin bacteria *Propionibacterium* to other Gram-negative bacteria, like *Pseudomonas* spp., and other lactic acid bacteria, such as *Enterococcus* spp. and *Weissella* spp., to name just a few; more details have been reported in the study of Jost et al. [223] and extensively reviewed in the meta-analysis of Fitzstevens et al. [224]. 

The most represented bacterial groups in HBM are *Staphylococcus* spp., *Streptococcus* spp., *Lactobacillus* spp. and *Bifidobacterium* spp. High inter-individual variability about the number and abundance of different species was evidenced in human milk; these different results between studies may be due to different sampling and process protocols and varied DNA extraction, selection of specific primers and sequencing platforms [199,200]. Moreover, in BM, there are many anaerobic and lactic acid bacteria, which could confer further anti-microbial protection and improve nutrients’ absorption [16]. 

Using shotgun amplification, Jiménez et al. identified a healthy core human milk microbiota that included seven genera: *Staphylococcus* spp., *Streptococcus* spp., *Bacteroides*, *Faecalibacterium* spp., *Ruminococcus* spp., *Lactobacillus* spp and *Propionibacterium* spp. [225]. Using NGS, Hunt et al. identified nine operational taxonomic units in all milk samples collected: *Staphylococcus* spp., *Streptococcus* spp., *Serratia* spp., *Pseudomonas* spp., *Corynebacterium* spp., *Ralstonia* spp., *Propionibacterium* spp., *Sphingomonas* spp. and *Bradyrhizobiaceae* [226]. 

Joining these two studies, HBM of healthy lactating women showed a unique microbial ecosystem with a dominant core of *Staphylococcus* spp., *Streptococcus* spp. and *Propionibacterium* spp. The core microbiota is composed of species needed for maintaining efficient ecosystem homeostasis whose loss (or gain) may negatively impact the structure and function of other members in the ecosystem [227]. Interestingly the core bacteria seemed to be less affected by the environmental factors (diet, obesity, stress) which influenced the composition of the other microbiota [228].

Pananraji et al. analyzed the bacterial composition in maternal breast milk, areolar skin and infant stool by sequencing of the 16S ribosomal RNA gene in order to estimate the contribution of the breast milk and areolar skin microbiota to the infant gut microbiota. The authors observed that during the first 30 days of life, infants who breastfed to obtain 75% or more of their daily milk intake received a mean of 27.7% of the bacteria from breast milk and 10.3% from areolar skin. Bacterial and composition diversity depended on proportion of daily breast milk intake [229]. 

The origin of breast milk bacteria is currently not known. Classically it was supposed that maternal skin and infant’s oral cavity represented the main source of HBM bacteria. The process, called retrograde flow during breastfeeding, explained that some bacteria derived from the transfer of oral and skin bacteria that enter the mammary ducts during suckling [230]. This theory is supported by the fact that *Streptococcus* spp., one of the major bacteria presented in HBM, also dominates the salivary microbiota. In addition, other common skin bacterial isolates, such as *Staphylococcus* spp., *Corynebacterium* spp. and *Propionibacterium* spp. [231,232] are frequently detected in HBM. Ultrasound imaging studies have shown that substantial retrograde flow occurs during the second half of milk ejection [230], which could be a plausible route for infant oral bacteria to enter the mammary ducts, as well as a potential pathway for exchange between the mammary gland and the infant’s oral cavity [233]. 

However, anaerobic genera found in HBM were not detectable on the skin [234], so another theory, called the entero-mammary pathway, was proposed. It has been hypothesized that maternal intestinal bacteria migrates from the maternal gut by internalization in dendritic cells and then circulates to the mammary gland via the lymphatic and blood circulation during pregnancy and lactation [235,236]. In support of this hypothesis, animal studies have shown increased bacterial translocation of both aerobic and anaerobic organisms from the gut to the mesenteric lymph nodes and mammary glands in pregnant and lactating mice [237] and similar butyrate-producing bacteria, including *Coprococcus* spp., *Faecalibacterium* spp. and *Roseburia* spp., have been detected in both maternal feces and human milk [218]. In addition, human breast tissue had a commensal microbiota, suggesting that specific microbes inhabit the breast tissue and potentially colonize the milk ducts [238]. As in HBM microbiota, the principal phylum, *Proteobacteria*, was the major phylum detected in human breast tissue microbiota [237]. 

Different factors like genetic factors, mode of delivery, maternal dietary habits and nutritional status, GA, lactation stage, the use of antibiotics or other medicine, maternal status and geographical region influence human milk composition and microbiota [239], as discussed below. 

A significant change in the composition of the breast milk microbiota has been observed over the lactation stage. The most common genera in colostrum samples detected by 16S sequencing included *Leuconostoc* spp., *Weissella* spp., *Staphylococcus* spp., *Streptococcus* spp. and *Lactococcus* spp. according to analyses of breast milk samples from 18 mothers from Finland. From 1 to 6 months after delivery, a significant increase was observed in *Veillonella* spp., *Prevotella* spp., *Bifidobacterium* spp., *Enterococcus* spp. and *Leptotrichia* spp. [240]. 

HBM microbiota depended on GA, with significant differences between term- and preterm-delivered mothers. HBM samples of mothers with term deliveries presented lower counts of *Enterococcus* spp. in colostrum and higher counts of *Bifidobacterium* spp. [241]. Interestingly, high microbial diversity and high prevalence of *Bifidobacterium* spp. and *Lactobacillus* spp. were detected in colostrum and milk following vaginal delivery, whereas the contrary was observed following CS [241]. Toscano et al. analyzed microbiota of colostrum by NGS of twenty-nine Italian mothers (15 vaginal deliveries vs. 14 CS). The authors evidenced numerous differences between CS and vaginal delivery colostrum; in particular, vaginal delivery colostrum seemed to have a significantly lower abundance of *Pseudomonas* spp., *Staphylococcus* spp. and *Prevotella* spp. compared to CS; instead, no differences were observed in terms of the count of anerobic bacteria genera. Interestingly, the colostrum of mothers who had a CS was richer in environmental bacteria than mothers who underwent vaginal delivery [242]. About mature milk, Cabrera-Rubio observed a higher bacterial diversity and richness in milk samples from vaginal deliveries in comparison to milk samples from CS; in particular, a higher relative abundance of *Staphylococcus* spp. and *Enterococcus* spp. and lower of *Streptococcus* spp. was found in CS milk samples. Quantitative PCR data evidenced that in all milk samples, higher levels of *Bifidobacterium* spp. were related significantly to lower levels of *Staphylococcus* spp. [243]. In addition, mothers who had elective CS also showed decreased members of the family *Leuconostocaceae* and increased *Carnobacteriaceae* compared with women who delivered vaginally [240], strengthening the role of delivery mode on HBM bacterial composition. 

However, no difference was observed between women who delivered vaginally and those who underwent emergency CS, suggesting that the stress and/or hormonal signals related to labor have an impact on bacterial transfer to the mammary gland [240].

In addition, maternal physiological status, including obesity, celiac disease and human immunodeficiency virus (HIV)-positive status are associated with changes in the HBM microbiota composition [240]. Obesity influenced levels of *Bifidobacterium* spp. and cytokines in human milk [87], as well as increased *Staphylococcus* spp., leptin and proinflammatory fatty acid levels [244] and reduced microbial diversity [240]. Samples of HBM of mothers with celiac disease presented lower levels of cytokines, *Bacteroides* spp. and *Bifidobacterium* spp. [245]. In addition, allergic women exhibited a significantly lower *Bifidobacterium* spp. in their HBM, and their infants were shown to have lower fecal *Bifidobacteria* counts [246]. Finally, HBM of African HIV-positive women presented higher bacterial diversity and prevalence of *Lactobacillus* spp. than HBM of women without HIV infection [247]. 

Soto et al. observed that perinatal use of antibiotics has an impact on the maternal microbiota, reducing the prevalence of *Lactobacillus* spp., *Bifidobacterium* spp. and *Staphylococcus* spp. [248].

Regarding geographical location, HBM microbiota evidenced in Spanish mothers were different from those in Americans [184]. In addition, Chinese women seemed to have high levels of *Actinobacteria* in comparison to the similarly high levels of *Bacteroidetes* detected in Spanish women [249]. Drago et al. analysed the microbiota network of colostrum and mature milk of Italian and Burundian mothers and observed that all samples showed different bacterial distributions in the microbiota network [250].

Further research is needed to fully understand the role of BM microbiota in the infant, the link between the milk microbiota and health benefit, the potential factors influencing this relationship and whether or not it can be influenced by nutrition, though the increasing evidence already highlights its importance on infants’ protection and training of the immune system during the first months of life.

## 5. Conclusions

Our review highlights how the neonatal microbiota can be affected before, during and after gestation, with a strong impact of maternal and environmental factors on the offspring outcome in the short- and long-term.

As highlighted, the gut microbiota is influenced by the type of birth, the place where it occurs, the mother’s state of health and the intake of antibiotics; mothers are the first to transmit their microbiota during gestation and vaginal birth and later through breastfeeding [193]. 

The microbiota undergoes changes throughout one’s whole life and establishes its symbiotic relationship with the host already in the womb.

For a better comprehension of these aspects, in the future, it will be necessary to also study the fetal response to thematernal environment, and it is desirable to integrate new techniques, such as omics technologies, able to characterize the metabolites of maternal and fetal origin, to clarify which are the real effectors. As discussed, many of the associations described to date should be reconfirmed in larger studies, all conducted with the same methodologies, due to the heterogeneity of the available studies in terms of number of recruited subjects, time of sampling and technologies applied. 

The greatest difficulties regarding the interpretation of the data are due to methodological limits, particularly of the methods of identification of the bacteria. The classic culture methods present cultivation difficulties observed with most of the microbes present in a microbial ecosystem, due to the difficulty of recreating the conditions/relationships existing in a microbial ecosystem. The new molecular methods avoid these difficulties but they have limits related to the type of reagents, to the possibility of contamination, and the inability to distinguish between live active and dead inactive bacteria. During the next few years, new knowledge on the functional aspects of the microbiota is expected, coming from metabolomics and proteomics [45,251].

The state of health of the mother before and after conception, the pregnancy outcome, the type and the place of delivery, neonatal treatment and neonatal nutrition through breastfeeding or formula milk seem to be the main factors that determine the establishment of neonatal microbiota, which can, in turn, determine positive or negative variations, potentially influencing the onset of several diseases such as NEC, SIDS, diabetes and asthma.

In this context, all pregnant women should be informed about the current evidence, being encouraged to follow a healthy lifestyle, promote their health and take care of their nutrition, through the knowledge of the benefits she could guarantee to her future child. The information of the benefits of reduced intrapartum antibiotics administration, the benefits of VAG delivery and those related to less neonatal pharmacological treatments, as well as the benefits of breastfeeding on neonatal microbiota, should always be taken into account by obstetrics and neonatologists. 

An adequate balance of neonatal microbiota at birth, starting from the womb, potentially determined by the discussed factors, could promote a positive short- and long-term effect not only on neonatal life but also in childhood and adulthood, improving health outcomes. Maternal–fetal–neonatal microbial interactions play the basis for a relationship that will last a lifetime.

## Figures and Tables

**Table 1:** Major bacterial taxa found at each colonization site of reproductive age women, and their impact on fertility, according to the studies discussed in the review. ART = assisted reproductive technique.

	Physiological	Bacterial Vaginosis	Infertility	ART Outcome
**Vagina**	- dominated by *Lactobacillus* spp. - Classified into five community state types (CST): CST I (*Lactobacillus crispatus* predominant), CST II (*Lactobacillus gasseri* predominant), CST III (*Lactobacillus iners* predominant), CST IV (non-*Lactobacillus* spp.). Type IV-A: low proportions of *Lactobacillus iners* or other *Lactobacillus* spp., various species of anaerobic bacteria including *Anaerococcus*, *Corynebacterium*, *Finegoldia*, or *Streptococcus*. Type IV-B: higher proportion of the *genus Atopobium*, *Prevotella*, *Parvimonas*, *Sneathia, Gardnerella, Mobiluncus, Peptoniphilus* and other taxa. CST V (*Lactobacillus jenseri* predominant) [28,36,37,38]	- Prevalence of *Gardnerella vaginalis*, *Mycoplasma hominis*, *Atopobium vaginale* and *Mobiluncus curtisii* [42,43,44,45] - higher bacterial diversity than physiological conditions [29,46]	- (*Chlamydia trachomatis*) ascending through the cervix [50,51,52,53] - higher percentage of *Lactobacillus gasseri, Veillonella* spp. and *Staphylococci* and lower content of *Lactobacillus iners* and *crispatus* [57]	- the diversity of bacterial species and the presence of *Lactobacilli* on the ET day improved the outcome [58]
**Uterus**	- *Lactobacillus iners*, *Lactobacillus crispatus*, *Prevotella* spp. [61]	-	- *Lactobacillus* spp. could improve fertility by inhibiting pathogenic bacteria [50,71]	- uterine microbiota lower in *Lactobacillus* spp., and non-*Lactobacillus* spp. dominated was associated with a lower ART success [60] and, on the contrary, *Lactobacilli* were associated with a negative impact ART outcome [62]

**Table 2:** Major bacterial taxa found during pregnancy and its complications, at each colonization site, according to the studies discussed in the review.

	Pregnancy
**Gut**	- especially in the third trimester, reduction in maternal gut microbiota diversity, with the increase of *Proteobacteria* [75,86], *Streptococci*, *Lactobacilli* [75] *Bifidobacteria* and species producing lactic acid [86] - in overweight women, reduction in *Bifidobacterium* spp. and *Bacteroides*, and increase in *Enterobacteriaceae, Staphylococcus* spp., *Escherichia coli* [84,87] - higher percentage of pathogenic bacteria, such as *Clostridium perfringens* and *Bulleidia moorei*, and a reduction in the *Coprococcus catus* in mothers affected by preeclampsia, while healthy controls were mostly characterized by *Bacteroidetes* spp. [88]
**Oral cavity**	- during the third trimester, increase in bacterial diversity and total amount [75,89,90]
**Vagina**	- progressive reduction in anaerobic bacteria and increase in *Lactobacillus* spp. [91,92]
**Placenta**	- prevalence of *E. coli* [5] - similarities with the oral microbiota [5] - *Lactobacilli*, *Propionibacteria*, *Enterobacteriaceae* [30] - in women who undergoing elective Cesarean section, lower diversity index and prevalence of *Proteobacteria* [93] - higher percentage of *Acinetobacter* spp. in women with gestational diabetes mellitus [94]

**Table 3:** Major bacterial taxa found in newborns at each colonization site, according to the studies discussed in the review.

	Newborns
**Oral cavity**	- *Streptococcus* spp. appears early after birth [5,11,180] - one week after birth, neonatal oral biofilm resembles that of the mother. The presence of anaerobic Gram negative *Fusobacterium nucleatum* is associated with maternal chronic periodontitis, and also with bacterial vaginosis and preterm delivery [10,181]
**Gut**	- most represented species in newborns < 33 weeks of gestational age: *Lactobacillus* spp., *Staphylococcus* spp., *Enterobacter* spp. and *Enterobacteriaceae* [122,141] - lower percentage of *Bacteroides* in the offspring of mothers following a high-fat diet - in the offspring of overweight mothers, increase in *Bacteroides* and reduction in *Enterococcus* spp., *Acinetobacter* spp., *Pseudomonas* spp. [20,144,145], and *Bifidobacterium* spp. [83,182] - following intra-partum antibiotics’ administration, decrease in bacterial diversity, reduction in *Actinobacteria* and *Bacteriodetes* and increase in *Proteobacteria* [13] - abnormal colonization by *Pseudomonas* spp. and *E. coli* was detected in necrotizing enterocolitis [183,184,185,186,187,188,189,190,191,192] - higher levels of *Clostridium difficile*, *Clostridium innocuum* and *Bacteroides thetaiotaomicron* in sudden infant death syndrome cases [193] - neonates born by vaginal delivery acquire microbial communities similar to maternal gut and vagina, while those born by cesarean section acquire environment-like bacteria, such as *Staphylococcus* spp., *Corynebacterium* spp. and *Propionibacterium* spp., are associated with lower microbial diversity and delayed colonization of *Bacteroides* spp. and *Bifidobacteri* [14]
**Meconium**	- most represented species: *Enterobacteriaceae, Enterococcus* spp., *Lactobacillus* spp., *Bifidobacterium* spp. [117,120] - very similar to the amniotic fluid [122] - in preterm infants, different microbiota than term infants [122,140,141], with lower bacterial diversity with the decrease in gestational age [122,140] - In preterm infants born by mothers with chorioamniotitis: large quantities of pathogenic bacteria, such as *Ureaplasma parvum, Fusobacterium nucleatum* and *Streptococcus agalactiae* [30] - in infants born by vaginal delivery, the abundance of *Bifidobacterium* spp. (7° and 14° day of life), *Bacteroides* spp. (7° and 14° day of life) and *Lachnospiraceae* spp. (7° day of life) was significantly greater than those born by cesarean section, with a lower abundance of *Enterobacteriaceae* spp. [194]

## Data Availability

not applicable.
